# Cell size control driven by the circadian clock and environment in cyanobacteria

**DOI:** 10.1073/pnas.1811309115

**Published:** 2018-11-08

**Authors:** Bruno M. C. Martins, Amy K. Tooke, Philipp Thomas, James C. W. Locke

**Affiliations:** ^a^Sainsbury Laboratory, University of Cambridge, CB2 1LR Cambridge, United Kingdom;; ^b^Department of Mathematics, Imperial College London, SW7 2AZ London, United Kingdom

**Keywords:** cell size control, single-cell time-lapse microscopy, cyanobacteria, circadian clock, stochastic modeling

## Abstract

How cells maintain their size has been extensively studied under constant conditions. In the wild, however, cells rarely experience constant environments. Here, we examine how the 24-h circadian clock and environmental cycles modulate cell size control and division timings in the cyanobacterium *Synechococcus elongatus* using single-cell time-lapse microscopy. Under constant light, wild-type cells follow an apparent sizer-like principle. Closer inspection reveals that the clock generates two subpopulations, with cells born in the subjective day following different division rules from cells born in subjective night. A stochastic model explains how this behavior emerges from the interaction of cell size control with the clock. We demonstrate that the clock continuously modulates the probability of cell division throughout day and night, rather than solely applying an on−off gate to division, as previously proposed. Iterating between modeling and experiments, we go on to identify an effective coupling of the division rate to time of day through the combined effects of the environment and the clock on cell division. Under naturally graded light−dark cycles, this coupling narrows the time window of cell divisions and shifts divisions away from when light levels are low and cell growth is reduced. Our analysis allows us to disentangle, and predict the effects of, the complex interactions between the environment, clock, and cell size control.

## Significance

When and at what size to divide are critical decisions, requiring cells to integrate internal and external cues. While it is known that the 24-h circadian clock and the environment modulate division timings across organisms, how these signals combine to set the size at which cells divide is not understood. Iterating between modeling and experiments, we show that, in both constant and light−dark conditions, the cyanobacterial clock produces distinctly sized and timed subpopulations. These arise from continuous coupling of the clock to the cell cycle, which, in light−dark cycles, steers cell divisions away from dawn and dusk. Stochastic modeling allows us to predict how these effects emerge from the complex interactions between the environment, clock, and cell size control.

Organisms control the size of their cells (1–5). In growing cell colonies or tissues, they must do this, in part, by deciding when to divide. The principles of cell growth and division in microorganisms have been studied for many years (6–8). Multiple size control principles have been proposed, including the sizer model, where cells divide at a critical size irrespective of birth size, or the timer model, where cells grow for a set time before dividing (9–15). Recent time-lapse analysis of microbial growth at the single-cell level suggested that many microorganisms follow an “adder” or “incremental” model (16–21), where newborn cells add a constant cell size before dividing again. This principle allows cell size homeostasis at the population level (15, 18).

Although the rules of cell division under constant conditions are being elucidated, cell division in many organisms is controlled by intracellular cues and time-varying environmental signals. For example, cell division and growth are tightly linked to light levels in algae (22–24), while growth is enhanced in the dark in plant hypocotyls (25). Earth’s cycles of light and dark can thus cause 24-h oscillations in cell division and growth. To anticipate these light−dark (LD) cycles, many organisms have evolved a circadian clock which drives downstream gene expression with a period of about 24 h (26). The circadian clock has been shown to be coupled to cell division in many systems, from unicellular organisms (27, 28) to mammals (29–31). It remains unclear how the clock modulates the innate cell growth and the division principles that organisms follow.

The cyanobacterium *Synechococcus elongatus* PCC 7942 is an ideal model system to address the question of how cell size homeostasis can be controlled and modulated by the circadian clock and the environment. Cell sizes are easily coupled to the environment as ambient light levels modulate growth (32), which can be monitored in individual cells over time (33–35). An additional advantage is that the key components of the circadian clock in cyanobacteria are well characterized. The core network consists of just three proteins (KaiA, KaiB, and KaiC) that generate a 24-h oscillation in KaiC phosphorylation (36–38). The state of KaiC is then relayed downstream to activate gene expression by global transcription factors such as RpaA (37, 39). Many processes in *S. elongatus* are controlled by its circadian clock (37, 39–41), including the gating of cell division (28, 35, 42). The prevalent idea is that cell division is freely “allowed” at certain times of the day (gate open) and restricted at others (gate closed).

Gating of cell division in *S. elongatus* was first described by Mori et al. (28) under constant light conditions. Their results indicated that cell division was blocked in subjective early night, but occurred in the rest of the 24-h day. Single-cell time-lapse studies under constant light conditions have further examined this phenomenon, and suggested a mechanism for it (35, 42). Elevated ATPase activity of KaiC has been proposed to indirectly inhibit FtsZ ring formation through a clock output pathway (42). Phenomenological models coupling the clock to the cell cycle have successfully captured properties such as the distribution of phases at division (35) or correlations between cell cycle durations in cell lineages (43). The maintenance of clock robustness during the cell cycle has also been studied using a mechanistic model (44). However, it remains unclear what effect the coupling of the clock to cell division has on cell size homeostasis for *S. elongatus*, and what are the underlying division rules.

**Fig. 1.**
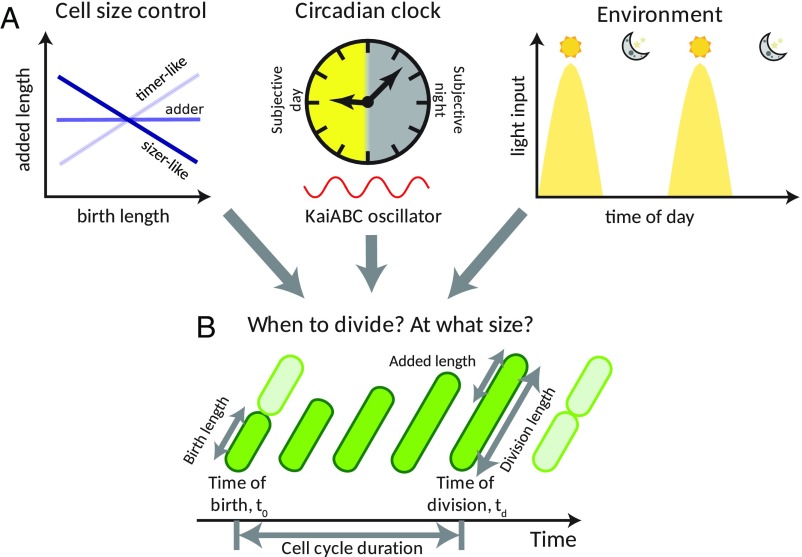
Multiple internal and external factors coordinate cell growth and divisions in cyanobacteria. (*A*) Cell division in cyanobacteria depends on cell-individual factors such as cell size control, the circadian clock, and the environment through light inputs. (*B*) To quantify the impact of these components on a cell’s decision to divide, we measured timings of birth and divisions and the increase in cell length, using time-lapse microscopy.

In this work, we examine how the environment and the clock modulate cell size control and the timing of division in *S. elongatus* (Fig. 1), using single-cell time-lapse microscopy. Under constant light conditions, the clock splits cells into two subpopulations following different size control and division rules. The specific properties of these subpopulations arise from modulation of cell size control by the clock throughout subjective day and night, rather than solely by repressing (gating) cell division in early night. Cells born during subjective night and early subjective day add less length before dividing again, allowing them to divide before the end of the day, while cells born during subjective day add more length, avoiding division in subjective night. We develop a stochastic model that explains these cellular decisions. To understand the significance of these results, we examine growth and division under realistic graded LD cycles. Combining modeling and experiment, we find that the clock narrows the window when cell division occurs. This prevents cell division from taking place at times when growth arrest could occur due to little or no light (45). Our predictive model reveals the contributions of the circadian clock, environment, and underlying cell size control mechanisms on division throughout the day and night.

## Results

### The Circadian Clock Generates Two Subpopulations Following Different Growth Rules Under Constant Light Conditions.

**Fig. 2.**
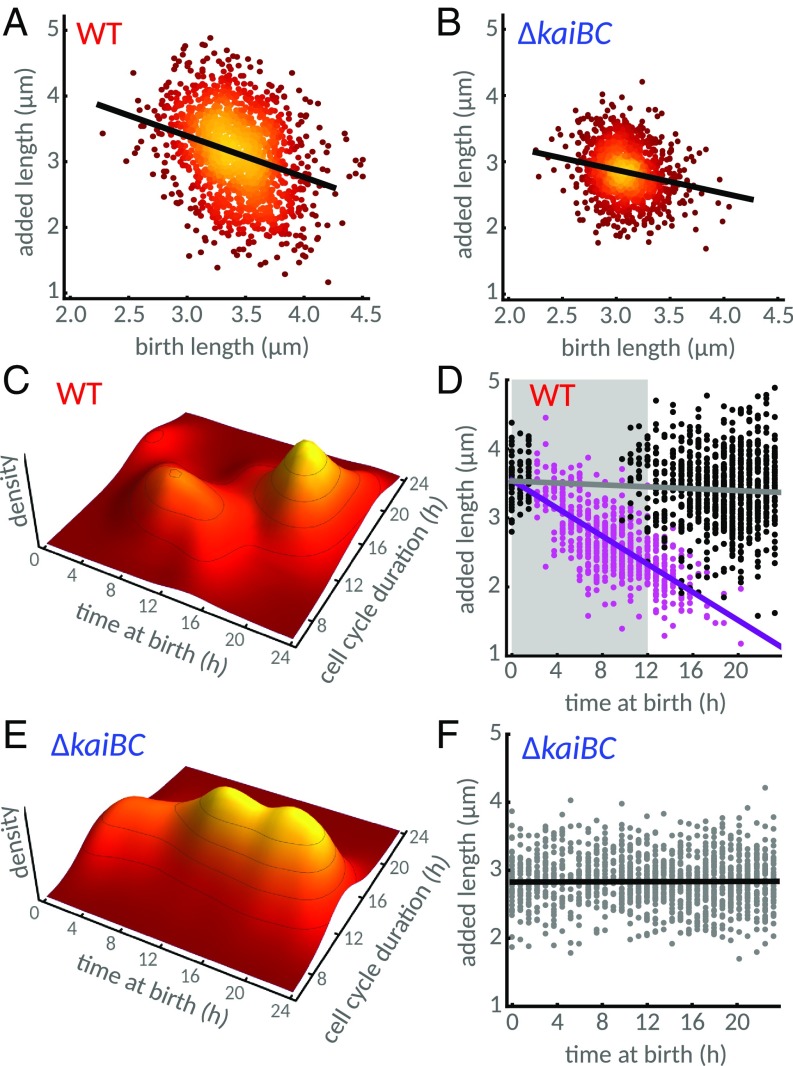
Coordination of cell size control by the circadian clock in *S. elongatus* generates two subpopulations under constant light. (*A*) WT cells follow an apparent sizer-like principle, where cells add shorter lengths the longer their birth length is (black line, linear regression with slope of −0.63); 1,529 individual cells from three independent experiments were analyzed. Lighter color indicates a higher density of points. (*B*) Cell size control in a clock-deletion mutant (Δ*kaiBC*) follows an adder-like principle more closely, with a weaker dependence of added length on birth length (black line, slope of −0.35); 1,348 individual cells from three independent experiments were analyzed. Color map is as in *A*. (*C*) The dynamics of WT cells exhibits two distinct subpopulations: Cells born late in subjective day have longer cell cycles than cells born earlier. (*D*) Clustering of the two subpopulations reveals an anticorrelation (slope of −0.1 μm⋅h^−1^, violet line) between time of birth and added length in cells born in late subjective night or early subjective day (magenta dots). These cells also add less length than the subpopulation of cells born during subjective day (black dots). Violet and gray lines show linear regression of the two subpopulations. The shaded gray area represents subjective night. (*E* and *F*) In populations of the clock-deletion cells, cell cycle durations and added length do not depend on the time of day at birth. Black line in *F* is the linear regression line.

To examine the role of the clock in cell size control in *S. elongatus*, we first studied growth and division in wild-type (WT) and clock-deletion backgrounds under constant light conditions. A clock-deletion strain (Δ*kaiBC*) was obtained by deleting the *kaiBC* locus, thus inactivating the KaiABC oscillator. We entrained cells under a regime of 12-h-light and 12-h-dark cycles (*Materials and Methods*), and carried out time-lapse microscopy movies under constant 15 μE⋅m^−2^⋅s^−1^ cool white light. We segmented and tracked thousands of individual cell lineages over multiple generations. The relation between size at birth and size added between birth and division is often indicative of the model controlling when cells decide to divide (15, 18). If size at birth is linearly related to added size with a slope of 1, then the underlying model is called a “timer,” in which cells wait a specific time before division. A slope of −1 is indicative of a “sizer,” where cells divide after reaching a critical size. More generally, negative slopes can be categorized as sizer-like, while positive slopes represent timer-like strategies (15, 46–48). Alternatively, added size may not correlate with size at birth (slope of 0). Such cells, which grow by a fixed size, irrespective of their birth size, are described as “adders” (16–18) (Fig. 1*A*). *Escherichia coli* and other microbes have been shown to obey this adder rule (15). *S. elongatus* cells are rod-shaped and grow in volume by increasing their pole-to-pole length, and so cell length is an appropriate measure of cell size (*SI Appendix*, section 1). Interestingly, in the WT background, *S. elongatus* cells are best fit by a sizer-like model (slope of −0.63), where the larger they are when born, the less length they need to add to reach a target length (Fig. 2*A*). This effect was less apparent in the clock-deletion background, where cells appeared to have a much weaker dependence on birth length (Fig. 2*B*) (slope of −0.35).

How can the circadian clock, which times processes to particular times of the 24-h day, cause cells to divide at a specific size? To address this question, we first examined how cell division is affected by the time of day. Time of day is determined over a length of 24 h, with a time of day of 12 h set at the end of the last dark period experienced by the cells during entrainment (see *SI Appendix*, section 3 for details). As has been reported previously (28, 35, 42), we observed apparent gating of cell division, with fewer cell divisions in the early subjective night in the WT (*SI Appendix*, Fig. S1*C*) but not in the clock-deletion background (*SI Appendix*, Fig. S1*A*). We next asked how this imbalance affects cell cycle durations. The distribution of cell cycle durations was not clearly bimodal (*SI Appendix*, Fig. S1*D*), but, by clustering cells based on time of day at birth and cell cycle duration (*SI Appendix*, sections 3 and 4 and Fig. S2), we found that cells lie in two distinct subpopulations (Fig. 2*C*). WT cells born either in late subjective night or early subjective day have shorter cell cycles (“fast” cells) than those born later in the day (“slow” cells). We also observed that the clock causes cells to divide faster at the end of subjective day and slower during other times, compared with clock deletion cells (*SI Appendix*, Fig. S1 *A*–*D*), suggesting that the clock can both repress and accelerate cell divisions.

Finally, the timing of cell division also affects added length. On average, cells born in late subjective night or early subjective day add less length (magenta dots in Fig. 2*D*), as expected from their shorter cell cycle durations (*SI Appendix*, Fig. S1 *C* and *D*). Interestingly, within this subpopulation, added length decreases with time of birth (violet line, Fig. 2*D*), allowing these cells to divide before the end of subjective day. By contrast, in the absence of the clock, no two subpopulations are apparent (Fig. 2*E*). This is because, in the clock-deletion strain, cell cycle duration does not depend on the time of birth, and added length is constant throughout the day (Fig. 2*F*). Taken together, our results provide an intuition for the role of the clock in modulating cell division, with the clock not solely enabling a checkpoint at the beginning of the night as previously proposed. The observation that cell cycle durations in the fast subpopulation decrease with time of birth (magenta dots, *SI Appendix*, Fig. S2*A*) suggests the clock actively promotes cell divisions during a narrow window before the end of the day.

### A Simple Model Explains the Coordination of Cell Size by the Circadian Clock.

How does the clock generate the observed complex relationship between added cell length, birth length, and time of day? Phenomenological models of cell size control usually assume a linear dependence between added length and birth length, which can be estimated by linear regression (Fig. 2 *A* and *B*). Models of this type have been used to quantify cell size control of microbes (15, 46, 48). However, a linear model alone cannot explain how the clock affects cell size control, e.g., the dependence of added size on time of day (Fig. 2*D*).

We therefore developed a model based on the modulation of the cellular division rate by the clock. This model assumes that the WT cellular division rate depends on three factors: (*i*) a clock-independent cell size control hazard $S\left(L,L_{0}\right),$ which quantifies the rate per unit length of triggering a division event (10, 49–51) in clock-deletion cells; (*ii*) a coupling function G(t) imposed by the circadian clock, a periodic function of the time of day t; and (*iii*) increase in cell length ∂L/∂t, leading to

$$\text{division rate}=\Gamma \left(L,L_{0},\frac{\partial L}{\partial t},t\right)=S\left(L,L_{0}\right)G\left(t\right)\frac{\partial L}{\partial t}.$$ [1]

The division rate thus depends on the instantaneous length, length at birth, growth rate, and time of day. In the following, we test the underlying assumptions of this model.

**Fig. 3.**
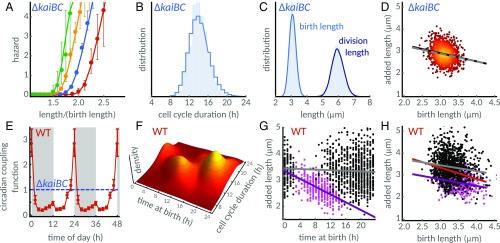
A simple model explains the emergence of the two subpopulations. Cell division rate is coordinated by growth rate, size control, and the circadian clock. (*A*) The cell size control hazard $S\left(L,L_{0}\right)$ estimated for clock-deletion cells (*SI Appendix*, section 6) with birth lengths between 2.4 μm and 2.8 μm (red points), 2.8 μm and 3.2 μm (blue points), 3.2 μm and 3.6 μm (yellow points), and 3.6 μm and 4.0 μm (green points) increases with length (error bars represent 95% bootstrap confidence intervals). The cell size control is explained by the linear model $S\left(\Delta_{0}\right)$ (lines). (*B* and *C*) Stochastic simulations (solid lines) of the model recover the observed cell cycle duration, birth length, and division length distributions (shaded areas). (*D*) Correlation between added length and birth length from simulations (solid black line, slope of −0.38) are consistent with the data (dashed gray line, slope of −0.35; compare with Fig. 2*B*). Scatter plot is obtained from numerical simulations, with lighter color indicating higher density of points. (*E*) Bayesian inference from single-cell traces (*SI Appendix*, section 7) reveals circadian modulation of cell divisions throughout the 24-h day in the WT. The coupling function G(t), parametrized by a positive periodic spline (red line, error bars denote 95% credible intervals at knots), decreases the division probability during subjective night (shaded gray background) and early subjective day (white background), compared with the clock-deletion background (dashed blue line). The coupling function, depicted for two cycles to highlight periodicity, peaks toward the end of subjective day. (*F*) Simulations using the inferred coupling function predict the emergence of two subpopulations as observed in the experimental data (Fig. 2*C*). (*G*) Clustering of the simulated data (subset shown) also predicts added length to decrease during the day in the fast subpopulation (violet line, slope −0.1 μm⋅h^−1^) but not in the slow population (gray line). Shaded gray area indicates subjective night. (*H*) The model predicts larger added cell lengths in the slow subpopulation (black dots) compared with clock-deletion cells, and smaller added cell lengths in the fast subpopulation (magenta dots). The respective slopes, which quantify the dependence of added length on birth length in the two subpopulations, are −0.30 (gray line) and −0.27 (violet line). The difference in cell size of the two populations explains the strong dependence of added length on birth length seen in WT cells (red line, slope −0.51).

To systematically disentangle the individual components affecting cell division rate, we first measured the size control $S\left(L,L_{0}\right)$ directly in clock-deletion cells, which do not gate or modulate cell divisions throughout the day [G(t)=1]. We find that size control in clock-deletion cells is indeed consistent with a size control hazard $S\left(L,L_{0}\right)=S\left(\Delta_{0}\right)$, which depends only on the birth-length−independent part of added length $\Delta_{0}$ (Fig. 3*A*). This implies a simple linear relationship $\Delta \left(L_{0}\right)=aL_{0}+\Delta_{0}$ between added length Δ and birth length $L_{0}$. The birth-length−independent part of added length $\Delta_{0}$ is a stochastic variable, and the parameter a quantifies the dependence of added length on birth length.

To parametrize the cell size control, we developed an unbiased parameter estimation method that accounts for unobserved growth before and after divisions (*SI Appendix*, section 6.3 and Fig. S3). Simulations of the resulting stochastic model with the same acquisition step as the experiments lead to discrete observation times. The simulations agree with the experimental distributions of cell cycle durations (Fig. 3*B*), with birth and division lengths (Fig. 3*C*), and also with the dependence of added length on birth length (Fig. 3*D*) in clock-deletion cells. We quantified the agreement using the means of these distributions, which match within experimental error bars (*SI Appendix*, Fig. S3).

Next, we verified the assumption that the circadian clock modulates the division rate independently of cell age or division length in *S. elongatus* using nonparametric estimations (*SI Appendix*, Figs. S4 and S5), which justifies the product form $S\left(L,L_{0}\right)G\left(t\right)$ of the division rate. In other words, the coupling function only depends on time of day t. We then used the model to estimate the circadian coupling function G(t) directly from individual cell length traces of WT cells via Bayesian inference. The method is based on the likelihood of divisions, which can be obtained analytically from Eq. **1** and is a function of the cell length history (*SI Appendix*, section 7). To avoid prior assumptions on the functional form of the coupling, we only require it to be a smooth, positive, and periodic function of the time of day.

Our analysis reveals that the circadian coupling function (Fig. 3*E*, red line) is at a low basal level throughout subjective night and early subjective day, effectively delaying cell divisions. Then, the coupling function progressively increases, peaking toward the end of subjective day, where it facilitates cell divisions compared with the clock-deletion strain (Fig. 3*E*, dashed blue line). We observe a similar dependence when estimating the coupling function using a simpler model (“division-time” model, *SI Appendix*, section 8) that only includes birth and division times (*SI Appendix*, Figs. S4 and S6). The additional information about cell length narrows the peak at the end of subjective day compared with the division-time model (*SI Appendix*, Fig. S6), and so it reveals a clearer separation between repression and promotion of cell divisions. All these different estimations of the coupling function (nonparametric, using the size-control model, and using the division-time model) share the same features, and provide quantitative support to the continuous modulation of divisions observed in the data (Fig. 2 *C* and *D* and *SI Appendix*, Figs. S1 *C* and *D* and S2*A*). The reduction in cell cycle duration as a function of time of birth we observed in the fast subpopulation (*SI Appendix*, Fig. S2*A*) can be attributed to the sharp peak in the coupling function at the end of the day, which increases the division rate.

Does our model of circadian modulation of size control (Eq. **1**) accurately predict the WT behavior? To determine this, we developed an exact simulation algorithm that can carry out detailed stochastic simulations of the model with the inferred time-dependent coupling function (*Materials and Methods*). To perform these simulations, we need to estimate the last term of the division rate, the rate of length increase, which can be computed from the single-cell length traces. We found that exponential elongation rates oscillate with a circadian period (*SI Appendix*, Fig. S7). This highlights that the circadian clock not only affects the decision to divide but also affects single-cell growth (45, 52). We incorporated the mean trend α(t) of these oscillations (*SI Appendix*, section 2 and Fig. S7) into the WT model.

In agreement with the experiments (Fig. 2*C*), the simulations reveal the emergence of differentially timed subpopulations with respect to their birth times (Fig. 3*F* and *SI Appendix*, Figs. S1 *E* and *F* and S2). We then asked whether the model could also explain the differences in size control observed in the two subpopulations. We verified that added length decreases throughout the day in the fast subpopulation (Fig. 3*G*, violet line with slope of −0.1 μm⋅h^−1^), in agreement with the experiments (Fig. 2*D*, violet line with slope of −0.1 μm⋅h^−1^), but not in the slow population (black dots and gray line, Figs. 2*D* and 3*G*). In contrast, when we forced the coupling function to take the form of a classical on−off gate, whether a piecewise square function (42) or a smoother continuous function (35), we were unable to capture these features of our data (*SI Appendix*, Fig. S8).

Interestingly, cells in the slow and fast subpopulations conform closely to the slope observed in clock-deletion cells, which is in agreement with experiments (see legend of *SI Appendix*, Fig. S9 for slopes and confidence intervals). The stronger dependence of added length on birth length seen in the overall WT population is thus an emergent phenomenon (red line, Fig. 3*H* and *SI Appendix*, Fig. S9). It arises from the differentially timed and sized subpopulations generated by the circadian clock.

### Environmental LD Cycles Combine with the Clock to Generate an Effective Coupling Function.

Like all other photoautotrophs, *S. elongatus* did not evolve under constant light. We therefore examined the effects of the circadian clock on growth and division under conditions more relevant to the natural environment of cyanobacteria. We grew WT and clock-deletion cells under graded 12-h-light and 12-h-dark cycles (12:12 LD) that approximate Earth’s cycles of daylight and dark (*Materials and Methods*). We programmed the light levels such that the flux of light per unit area over a period of 24 h is identical in constant light and in 12:12 LD.

**Fig. 4.**
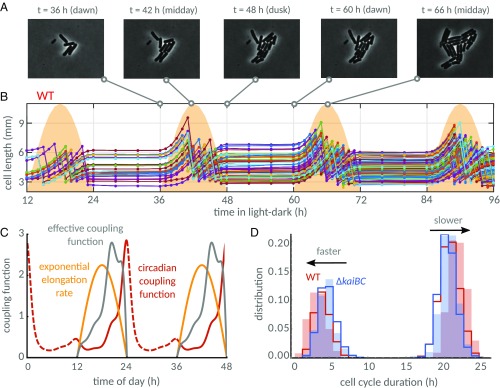
Under LD cycles, the combined effects of the environment and the clock on cell division generate an effective coupling of the division rate to the time of day. (*A*) Phase contrast images of a WT microcolony under graded 12:12 LD cycles reveal periods of growth followed by periods of stagnation between dusk and dawn. (*B*) Length profile of single cells (colored lines; each line represents an individual cell or lineage) in a time-lapse movie (some cells not shown in phase contrast images in *A* are also plotted). Cell growth and divisions are suspended in the dark and slowly rise when the lights turn on. The yellow shades indicate the light profile imposed on cells during the experiment (maximum at midday is ∼47 μE⋅m^−2^⋅s^−1^). (*C*) Exponential elongation rates of WT (yellow line) and clock-deletion cells closely follow the imposed light profiles (*SI Appendix*, Fig. S10). Exponential elongation rate (yellow) and circadian clock (constant light coupling function in red) impose an effective coupling function (gray line; product of exponential elongation rate and the circadian coupling function, Eq. **2**), shifting the probability of cell division away from dawn. (*D*) The light conditions split cells into two subpopulations: those that complete a full cycle when the lights are on (short cell cycle durations) and those that must wait until the next day to complete a cell cycle (long cell cycle durations). Our model shows that the fast WT population (red line) is faster than in the clock-deletion mutant (blue line), but the slow subpopulation is faster in the clock-deletion background. This prediction is confirmed by experimental histograms (background shades) of cell cycle durations for WT (1,009 cells from two experiments) and clock-deletion cells (1,201 cells from two experiments).

There was no visible growth or cell divisions in the dark (Fig. 4 *A* and *B*). As such, the pattern of LD cycles controls the growth rate of both WT and clock-deletion cells. Growth rates are also set by the level of ambient light during the day. In graded LD cycles, the mean exponential elongation rates of the two strains are nearly identical, and both track the level of ambient light quite accurately (Fig. 4*C* and *SI Appendix*, Fig. S10) (53). Restricting growth also constrains the distribution of cell cycle durations, separating cells into two subpopulations: cells that divide in the same day they were born (fast cells) and cells that divide only the day after they were born (slow cells) (Fig. 4*D*). This effect was observed in both WT and clock-deletion cells.

We therefore wondered how the circadian clock interacts with growth cycles imposed by the ambient light levels. We readjusted the size control term, by estimating size control parameters from clock-deletion data in LD conditions (*SI Appendix*, section 9 and Fig. S11), and imposed the time-dependent exponential elongation rates onto our model of WT and clock-deletion cells. We used the same coupling function estimated by Bayesian inference from the size control model in constant light (Fig. 3*E*). We adjusted the state of the coupling function at dawn to match subjective dawn in constant light (12-h mark in Fig. 3*E*; see also *SI Appendix*, section 3), representing the effect of the entrainment of the circadian clock in the model. We found that the clock accelerates cell divisions in the fast subpopulation but, interestingly, delays divisions in the slow subpopulation (blue and red lines, Fig. 4*D*). In qualitative agreement with this result, we found, experimentally, that cell cycle durations of the two subpopulations were shifted in opposing directions (blue and red shades, Fig. 4*D*).

These findings are explained through an effective coupling of the division rate to the time of day. Because mean exponential elongation rates α(t) track the level of ambient light I(t) during the day (*SI Appendix*, Fig. S10), the division rate is modulated in a time-dependent manner, not just by the circadian coupling function G(t), but also by the environment via cell growth α(t)=α(I(t)). This modulation imposes an effective coupling function in our model,

effective coupling function=G(t)α(t), [2]

which reflects the time-dependent part of the division rate. It implies that the division rate in clock-deletion cells [G(t)=1] is temporally modulated by growth rate. In comparison with clock-deletion cells, effective coupling also accounts for modulations by the clock (Fig. 4*C*) that delay cell divisions at dawn but accelerate division close to dusk in the WT (Fig. 4 *C* and *D*). This causes divisions in the fast subpopulation to accelerate, but it delays divisions in the slow subpopulation, as cells born the previous day would otherwise divide closer to dawn, highlighting the predictive power of our model (Fig. 4*D*). Next, we answer the question of what role the clock plays in size control in LD cycles.

### The Circadian Clock Modulates Cell Size in LD Cycles.

**Fig. 5.**
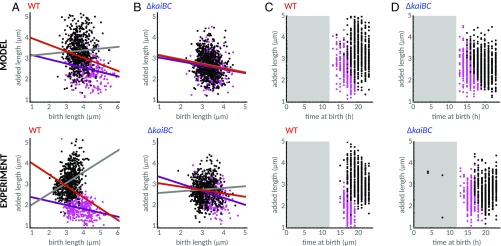
The circadian clock modulates the size control in 12:12 LD cycles. Model predictions (*Top*) and experimental data (*Bottom*). (*A*) The model (*Top*) predicts that the two subpopulations of cells in the WT (short cell cycles in magenta and long cell cycles in black) obey different cell size control strategies. Fast dividing cells obey an adder-like principle (violet line from linear regression, slope of −0.2), while slow dividing cells show a timer-like size control (gray line, slope of +0.1). This trend is also observed in the experiments (*Bottom*; violet line slope of −0.2, gray line slope of +0.5). Red line is the overall regression line when ignoring the presence of two subpopulations. (*B*) In the clock-deletion population, slow and fast cells obey similar trends in size control compared to the overall population trend. (*C*) Fast cells (magenta dots) are born earlier in the day on average, and add less length than cells whose cell cycle spans to the next day (black dots). (*D*) In contrast to the WT, in the clock deletion mutant, both subpopulations increment approximately the same length to their birth lengths. Gray shades in *C* and *D* indicate darkness (lights off). Experimental sample sizes as in Fig. 4.

To understand the effect of varying light levels on cell size control, we used the model to predict the type of cell size control in the two subpopulations in LD cycles. Our model predicted that WT cells with short cell cycles, i.e., cells that are born and divide within the same day (magenta dots in Fig. 5 *A* and *C*, *Top*), add roughly half the length of cells with longer cell cycles, i.e., cells that divide a day after they were born (black dots, Fig. 5 *A* and *C*, *Top*). Whereas in constant light, slow cells, with longer cell cycle durations, grow larger on average, such a supposition is not necessarily true in LD conditions. This is because slow cells typically also live through the lowest light levels, i.e., the least favorable conditions. Indeed, no difference in added length between the two subpopulations is predicted for the clock-deletion mutant (Fig. 5*B*). The dependence of added length on subpopulation type and the differences between the two strains are confirmed by the experiments (Fig. 5 *A* and *B*, *Bottom*).

Furthermore, the model suggests that cell size control obeys different rules in the two dynamical subpopulations. Fast dividing cells increment their length with a weak dependence on birth size (Fig. 5*A*, violet line), similarly to the behavior of the clock-deletion strain in constant light. On the other hand, added length of slow cells increases with birth size (gray line), i.e., a timer-like size control, which was also confirmed by experiments (Fig. 5*A*, *Bottom*). This timer-like phenomenon is explained by the effective coupling function, which lowers the division rate in the early hours of the day (Fig. 4*C*). In effect, slow cells that were born in the previous day (before darkness) have to delay divisions until later in the day, which originates the timer-like behavior. Clock-deletion cells, on the other hand, do not display significant differences in cell size and cell size control between the two subpopulations (Fig. 5 *B* and *D*).

### The Effective Coupling of Divisions to the Environment and the Clock Modulates the Frequency of Cell Divisions in LD Cycles.

**Fig. 6.**
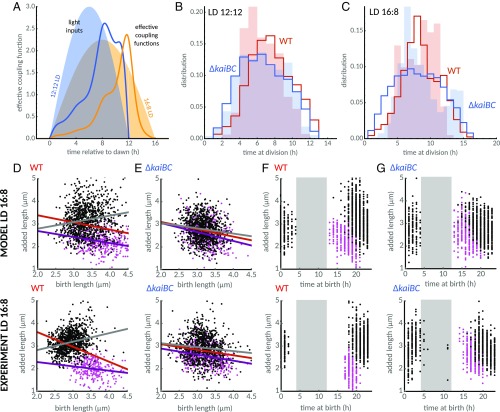
The circadian clock steers cell divisions away from dawn and dusk. (*A*) Imposed light levels (blue and yellow shades) interact with the circadian clock to create an effective coupling function (solid lines). For 12:12 LD cycles, the effective coupling function suggests that cell divisions occur away from dawn, while, in 16:8 LD cycles, divisions are shifted away from dawn and dusk. (*B* and *C*) Division time distributions of WT (red) and clock-deletion cells (blue) obtained from experiments (shades) in two LD conditions are compared with model predictions (solid lines). WT distributions are tighter than clock-deletion distributions. Experimental sample sizes for 12:12 LD as in Figs. 4 and 5. For 16:8 LD, we analyzed 1,226 WT cells from two experiments, and 1,372 clock-deletion cells from two experiments. (*D* and *E*) Model predictions (*Top*) and experimental data (*Bottom*) of the relation between birth length and added length for (*D*) WT and (*E*) clock-deletion cells under 16:8 LD conditions. As under 12:12 LD cycles, both the model and experiments show two subpopulations of cells obeying different cell size control strategies in the WT [cells that were born and divided in the same day (fast cells): magenta dots and respective violet line from linear regression; cells whose cell cycle spans over a dark cycle (slow cells): black dots and gray regression line]. Red line shows the linear regression over the whole population. In clock-deletion cells (in *E*), both subpopulations behave similarly. (*F*) Under 16:8 LD conditions, fast cells (magenta dots) in WT are born earlier in the day, on average, and add less length than slow cells (black dots). (*G*) In the clock-deletion mutant, both subpopulations increment approximately the same length over a cell cycle. Gray shades in *F* and *G* indicate darkness (lights off). The range of the darkness is 4 h to 12 h, which reflects the fact that, in 16:8 LD cycles, we extended “daylight” by adding 4 h of light at the end of day relative to the 12:12 LD regime. In other words, for two experiments run in parallel (12:12 LD and 16:8 LD), dawn occurs simultaneously in the two experiments, but dusk occurs 4 h later in 16:8 LD.

We next asked whether the clock affects the time at which cells are born in graded LD cycles. In the absence of a clock, G(t)=1, and so ambient light effectively dictates the division rate (Fig. 6*A*, Eq. **2**, and *SI Appendix*, Fig. S10*B*). In WT cells, however, our model predicts fewer cell divisions at dawn and a narrower distribution of division times, which results from the profile of the effective coupling function (Fig. 6*A* and Eq. **2**). This prediction is confirmed by the experimental distributions (Fig. 6*B* and *SI Appendix*, section 5). WT cells do not divide immediately after dawn, and wait longer than cells in the clock-deletion strain (Fig. 6*B*). Specifically, we find that 90% of WT cells divide within a window of 4 h to 10 h (model prediction: 3 h to 11 h) after dawn compared with 2 h to 11 h (model: 2 h to 11 h) for the clock-deletion mutant.

To test our understanding of this effect, we interrogated the model under prolonged light durations. In graded 16:8 LD cycles, the effective coupling function suggests that the presence of the clock would cause fewer divisions at dawn and dusk, as this effective function peaks closer to midday (Fig. 6*A*). To test this prediction, we repeated the experiment in a 16:8 LD condition. First, we confirm that the clock affects cell size control similarly to the 12:12 LD condition (Fig. 6 *D*–*G*). In WT and clock-deletion backgrounds alike, cells exhibit slow and fast dividing subpopulations. However, owing to the clock, it is only in WT that these two subpopulations exhibit different cell sizes and size control rules (Fig. 6 *D*–*E*). The experiments further confirm that WT cell divisions occur in a much narrower range of the day (90% of cells divide between 5 h and 12 h after dawn; Fig. 6*C*) than for the clock-deletion mutant (4 h and 14 h after dawn), in agreement with the theoretical conclusions drawn from the effective coupling function (Fig. 6*A*).

## Discussion

In this work, we used single-cell data and systematic interrogation of a stochastic model of cell growth to elucidate how the circadian clock and the environment rework underlying rules of cell size control in *S. elongatus* (Fig. 1). We first characterized cell size control in constant conditions and found that the clock generates an apparent sizer-like behavior (Fig. 2*A*). We showed that this effect is an epiphenomenon caused by the clock generating two subpopulations following different division rules in WT cells (Fig. 2 *C* and *D* and *SI Appendix*, Fig. S9). These subpopulations differ in cell cycle duration, added size, and times of birth and division relative to a 24-h day, while no such coordination is present in clock-deletion cells (Fig. 2 *E* and *F*). These results show that organisms possessing clocks, or coupling their cell cycle to intracellular or extracellular processes that drive them out of steady state, could have complex size control rules. These can even include more than a single type of cell size control for the same growth condition.

We then formulated a phenomenological model of how the circadian clock coordinates cell size control and cell division rate. The model confirmed that this interaction indeed generates two differently timed and sized subpopulations. Statistical inference using constant light data revealed that the clock modulates cell divisions by progressively increasing the division rate just before subjective dusk, while decreasing it at other times of the 24-h day (Fig. 3*E* and *SI Appendix*, Fig. S6).

Under graded LD cycles, the model predicted that the clock accelerates divisions in the fast subpopulation but delays divisions in the slow subpopulation, a finding that we confirmed experimentally under natural 12:12 LD cycles. By doing so, the clock constricts the time window of cell division. This effect was even more apparent, in both the model and experiments, under a 16:8 LD cycles condition, where divisions are driven away from dawn and dusk. Such a modulation of the timing of cell divisions could provide a fitness advantage by, for instance, avoiding cell division during the energetically unfavorable periods around dawn and dusk. Previous work suggested that the circadian clock’s slowing of growth rate before dusk can aid individual cell survival (45). In future, it will be important to investigate whether the clock’s restriction of cell division toward the middle of the day plays a similar functional role.

By inferring the coupling function between the clock and the cell cycle under constant light conditions, we revealed the qualitative features of clock control of cell size in *S. elongatus*. The clock progressively increases the probability of division throughout the second half of subjective day. The probability of division reaches a well-defined peak, threefold higher than the clock-deletion reference (Fig. 3*E*), just before dusk, before dropping to a basal level after dusk. This adds to our understanding of how the clock controls the cell cycle, revealing that the probability of division is under continuous circadian regulation.

Previous studies have proposed that the clock gates cell division by imposing a closed gate in the early hours of subjective dark, and thus causing the scarcity of cell division events observed during that window (28, 35, 42). In our model, this scarcity is generated by the lower level of the coupling function after dusk, but also by its sudden relaxation back to its basal level following the “rush” of divisions before dusk (Fig. 3*C*). This peak in the division rate can be interpreted as imposing an effective gate in the hours that immediately follow it. However, we observe that the peak in the coupling function also generates a progressive decrease in added size toward subjective dusk, which is not predicted by a classical two-level (on and off) gating function (*SI Appendix*, Fig. S8). To further validate our results, we applied our Bayesian approach, which does not constrain the type of coupling function, to an existing dataset obtained by Yang et al. (35). Both our dataset and that of Yang et al. share similar features (*SI Appendix*, Fig. S12), namely, a peak of cell division toward the end of the day and lower division rates at other times. Our findings thus explain the dependence of cell size on the time of birth.

Remarkably, the coupling function, fitted just on constant light data, accurately predicts the effects of the clock on cell size in both 12:12 LD and 16:8 LD light conditions. These predictions include the reduction of cell cycle durations for cells that divide in the same day they were born, and the increase of cell cycle duration for cells that divide in the next day. We elucidate that these complex phenomena can be understood through an effective coupling function accounting for the clock and environment. Our understanding of these nontrivial effects could help reveal the clock’s function in controlling cell division. In this respect, it will be critical to understand the molecular mechanism behind this coupling function in future work. One possibility is that the mechanism could be a combination of the repressive effects of KaiC ATPase activity on the cell cycle (42) with circadian activation of cell cycle control genes. For example, FtsZ expression is under circadian regulation, peaking near dusk (54). In summary, by incorporating statistics of both cell size and division times, our findings shed light on how the circadian clock governs a cell’s decision to divide at different sizes.

Although our simple coupling function reproduces most of the qualitative features of the cell size control and division rules observed experimentally, in future, it would be interesting to construct a more refined model, and to examine the few observations our model could not explain. For example, in this study, we did not consider how the duration of the light period under LD cycles may affect clock phase. Recent work by Leypunskiy et al. (55) found that, under discontinuous (on−off steps) LD cycles, the clock eventually entrains to track midday. By contrast, in our model, we reset the clock at dawn, such that the coupling function always peaks ∼12 h after dawn in both 12:12 and 16:8 LD cycles. Leypunskiy et al.’s results suggest the coupling function could have a temporal offset in 16:8 LD, peaking 2 h later relative to 12:12 LD, which could explain the offset between the peaks of the experimental and simulated distributions in Fig. 6*C*. Cell size control is also modulated by light conditions (*SI Appendix*, Fig. S11 *A*–*F*). Presently, the cell size control has to be reparametrized for each condition (constant light, 12:12 LD, and 16:8 LD cycles). Although this enables us to examine the role of the clock in cell size control, it will be important to develop a more complete model of how cell size control in the clock deletion strain is modulated by growth conditions (18, 51, 56). This might shed light on why the effective coupling function model does not predict the distribution of division times in clock-deletion cells under 16:8 LD cycles, which does not seem to follow the light levels imposed throughout the day (blue shades, Fig. 6*C*), as it does in 12:12 LD cycles. It would be interesting to capture these effects by extending our model through further iteration between experiment and theory.

Examining the relationship between added cell size and birth size has provided valuable insights into how microbes maintain cell size (15–18, 21). However, cells are subject to internal or external cues, which can affect cell size control in nonintuitive ways. In other organisms, including higher eukaryotes, cell division is also subject to a range of internal and external inputs, including the circadian clock. In future, it will be critical to develop predictive models of how these inputs feed into the regulation of cell physiology in these other organisms, similar to what we have done here. As we showed, such models provide unprecedented insights by disentangling the components affecting cellular decision-making.

## Materials and Methods

### Strains, Plasmids, and DNA Manipulations.

*S. elongatus* WT was obtained from an ATCC cell line (ATCC 33912). A clock-deletion strain was generated by insertion of a gentamycin resistance cassette into the ORF of the *kaiBC* operon. The plasmid (a gift from Erin O’Shea, Janelia Research Campus, Howard Hughes Medical Institute, Ashburn, VA), carrying the interrupted gene with the antibiotic selection marker, was transformed into the WT strain through homologous recombination. Complete allele replacement on all of the chromosomal copies was checked through PCR. Strains and plasmid used in this study are listed in *SI Appendix*, Table S1.

### Growth Conditions.

The strains were grown from frozen stocks in BG-11 media at 30 °C under photoautotrophic conditions with constant rotation. The Δ*kaiBC* strain was supplemented with gentamycin at 2 μg⋅mL^−1^. Light conditions were maintained at ∼20 μE⋅m^−2^⋅s^−1^ to 25 μE⋅m^−2^⋅s^−1^ by cool fluorescent light sources. Before the start of each movie acquisition, all cultures (whether they were used in experiments under constant light or in experiments under LD cycles) were kept at exponential phase, and entrained by subjecting the cells to a 12:12 LD.

### Microscopy and Sample Preparation.

A Nikon Ti-E inverted microscope equipped with the Nikon Perfect Focus System module was used to acquire time-lapse movies of the cells over several days. Two microliters of entrained cultures in exponential phase were diluted to an OD_750_ of 0.15 to 0.20 and spread on agarose pads. The agarose pads were left to dry and then placed inside a two-chambered coverglass (Labtek Services), which was brought under the microscope. To minimize effects of the sample preparation protocol on entrainment, cells in all experiments (constant light and LD cycles) were placed on the pads during the light period of the 12:12 LD entraining cycle from liquid culture, and were then maintained in the 12:12 LD regime under the microscope until the end of the 12-h dark period. Image acquisition began after the lights were turned on (dawn). Illumination for photoautotrophic growth was provided by a circular cool white light LED array (Cairn Research), attached to the condenser lens of the microscope. Light conditions were preprogrammed to run during acquisition. The setup allowed for instantaneous and near-continuous light level updates. In constant light experiments, the light level was set at ∼15 μE⋅m^−2^⋅s^−1^. In experiments with LD cycles, light was set such that the flux of photons per unit area was the same as in constant light over a 24-h period. The daily profile of solar insolation in the wild was approximated by the function

$$I\left(t\right)=\{\begin{array}{c} I_{max}\\ 0 \end{array}\begin{array}{c} \sin\left(\frac{2\pi mod\left(t_{exp}-12,24\right)}{2T_{L}}\right) \end{array}\begin{array}{c} \text{if}\;0\leq mod\left(t_{exp}-12,24\right)\leq T_{L},\\ \text{otherwise}, \end{array}$$

where mod is the modulo operator, $T_{L}$ is the duration of the light period (12 h or 16 h), *t_exp_* is time relative to the start of the experiment, and $I_{max}=24\;A\pi /2T_{L}$. A ≈ 15 μE⋅m^−2^⋅s^−1^ is light level in constant light, and so $I_{\max}$ ≈ 47 μE⋅m^−2^⋅s^−1^ in 12:12 LD. Data acquisition was controlled through the software Metamorph (Molecular Devices). At each time point, phase contrast and fluorescent images using the filter set 41027-Calcium Crimson (Chroma Technology) and a CoolSNAP HQ2 camera (Photometrics) were acquired. The fluorescent image was acquired with low exposure time and low excitation intensity in order not to disturb cell growth, and was used to improve image segmentation. Further, we did not examine cells carrying fluorescent reporters, to avoid any potential effects of phototoxicity on growth rates and thus on cell size. In constant light, images were acquired every 45 min. In LD conditions, images were acquired every hour during the day. The reduction in the frequency of acquisition was implemented to avoid phototoxicity when the light levels are very low and growth is slow. The frequency of acquisition was therefore further reduced in the dark. Finally, the light levels were updated after each stage position was visited and acquired. In between time points, the light levels were updated every 2 min. All experiments used a 100× objective. This protocol was adapted from Young et al. (57).

### Exact Stochastic Simulation Algorithm Coupling the Circadian Clock to Cell Size Control.

We provide an exact simulation algorithm to simulate a lineage of an exponentially growing and dividing cell from $t_{0}$ to T with division rate Γ (Eq. **1**). The simulation uses a thinning method (58, 59) with a time horizon Δt over which the division rate Γ is bounded by a constant B. For this purpose, we assume that (*i*) the function S is monotonically increasing and (*ii*) the coupling function and mean exponential elongation rate are bounded, such that $G_{max}\geq sup_{t}G\left(t\right)$ and $\alpha_{max}\geq {sup}_{t}\alpha \left(t\right)$, respectively. We simulate cell divisions within discrete observation times acquired every Δt: When a division occurs, division lengths are recorded in the previous step, while birth lengths are recorded in the next step.

### Algorithm.

 **Initialize** $t\leftarrow t_{0}.L\left(t\right)\leftarrow L_{0}.\;\zeta \leftarrow \zeta_{0}.$

**While** t≤T:

 1. Simulate L(t) from t to t+Δt using dL/dt=ζα(t)L. (cell growth)

 2. Draw an exponentially distributed random variable τ with rate

  $B=S\left(L\left(t+\Delta t\right)-\left(1+a\right)L_{0}\right)G_{max}\;\zeta \;\alpha_{max}L\left(t+\Delta t\right)$.

 3. **If** τ>Δt:

  Update t←t+Δt.

 4. **Else**:

  Update t←t+τ.

  Evaluate Γ(t) and draw a uniform random variable u.

  **If** Γ(t)≥Bu:

   Update L(t)←L(t)/2, $L_{0}\leftarrow L\left(t\right)$ and ζ←Normal(1,σ) (cell division)

We assume that cells grow exponentially, where α(t) is the mean instantaneous exponential elongation rate (*SI Appendix*, Figs. S7 and S10), and ζ is a random Gaussian distributed amplitude with SD σ that fluctuates from cell to cell. The parameter values used in the simulations and further details can be found in *SI Appendix*, section 9.

### Data Availability.

Single-cell data on both times and lengths at birth and division are available at the Cambridge University DSpace Repository (https://doi.org/10.17863/CAM.31834).

## Supplementary Material

Supplementary File
