# Supplementary material for: Cell size control driven by the circadian clock and environment in cyanobacteria
Source: Proc Natl Acad Sci U S A. 2018 Nov 8;115(48):E11415–24. doi: 10.1073/pnas.1811309115 (PMC6275512; doi:10.1073/pnas.1811309115)
Supplement: Supplementary File [file pnas.1811309115.sapp.pdf]

PNAS
www.pnas.org

Supplementary Information for

Cell size control driven by the circadian clock and the environment in cyanobacteria

Bruno M.C. Martins, Amy K. Tooke, Philipp Thomas, James C.W. Locke

Philipp Thomas
Email: p.thomas@imperial.ac.uk

James C.W. Locke
Email: james.locke@slcu.cam.ac.uk

This PDF file includes:

Supplementary text
Figs. S1 to S12
Table S1
References for SI reference citations

www.pnas.org/cgi/doi/10.1073/pnas.1811309115

Supplementary Information Text

1. Segmentation and cell length

All images were segmented and tracked using a modified version of Schnitzcells (1). The segmentation algorithm is performed on combined fluorescent and phase contrast images. The fluorescent image is acquired in the range of red wavelengths, and so it collects the cells' auto-fluorescence. Since *S. elongatus* cells are rod shaped, cell size is proportional to cell length. Cell length is defined as the length of the semi-major axis of the segmented cell shapes.

Only cells that divided within 120 h of the start of the experiment were used in the subsequent analysis.

2. Exponential elongation rates

Time-series of cell length were extracted and smoothed with either a locally weighted regression (lowess) method (for constant light data) or a moving average window 3 data points wide (LD cycles). We defined the exponential elongation rate as

$$\frac{\log(L_j) - \log(L_{j-1})}{t_j - t_{j-1}},$$

where L_j is the smoothed cell length at time t_j.

3. Defining time of day in constant light and in LD cycles

In all experiments, cells were entrained by 12:12 LD cycles (step cycles) before image acquisition. Clock phase is reset after prolonged periods of darkness (2), and the clock maintains robust and synchronous oscillations at the single-cell level for long periods, even under constant light (3). For simplicity, we assume that a circadian cycle lasts 24 h, and we define 'time of day' (t in the model), as a proxy for clock phase, in relation to environmental transitions: dawn (when lights are switched on) and dusk (when lights are switched off). Times of day are therefore set at the beginning of the experiment, and reset every 24 hours. Under constant light, subjective dawn occurs 24 h after the end of the last 12 h long dark period, and every 24 h thereafter. Subjective dusk occurs 12 h after subjective dawn. Times of day relative to a 24-hour day are thus obtained by shifting total experimental time t_{exp} by $(t_{exp} - 12) \text{ modulo } 24$.

In light-dark cycles, times of day at dawn are the same for 12:12 LD and 16:8 LD conditions. Under 16:8 LD cycles, the light period is extended by 4 h at the end of the day relative to 12:12 LD, and dusk occurs at a time of day $t = 4$ h.

4. Subpopulation clustering

For each tracked single cell that completed a cell cycle during image acquisition, we extracted distributions of time of birth, time of division, length at birth, length at division, added length and cell cycle duration. Joint distributions of time of birth and cell cycle duration under constant light revealed two distinct peaks (Fig. 2C). Both peaks are approximately symmetric and bell shaped along each dimension, so we assumed they can be fitted with Gaussian distributions, and that all points can therefore be thought of as belonging to a probabilistic mixture of two Gaussians (clusters).

The mixture components yield the likelihood that a measurement belongs to either cluster. We then applied the likelihood-ratio criterion to assign cells to either of the two clusters or subpopulations (see Supp. Fig. 1 for results from experiment and simulation). For light-dark data, the data falls naturally into two well separated subpopulations, which are defined according to whether the cell cycle history of a cell included a period of darkness or not, so in principle no mixture model is needed.

5. Analysis of division times using lineage-weighting

To analyse the frequency of cell divisions throughout the day, we used a lineage-weighted estimation in Fig. 6B,C. To this end, we extracted all lineages from the data and weighted each data point by a factor $1/2^D$ depending on the number of cell divisions D in that lineage. The method takes into account that fast growing cells are overrepresented compared to slow growing ones (4–6). We found the difference to the unweighted data is significant only in the LD conditions, because the two subpopulations have vastly different interdivision times.

6. Estimating cell size control parameters in the clock-deletion strain

6.1 Nonparametric estimator of the size control hazard

The relation between the division rate Γ (with $G(t) = 1$) and the cell cycle duration distribution is

$$\mathrm{P}(\tau|L_0) = \Gamma(L(\tau), L_0, \partial L/\partial\tau)e^{-\int_0^\tau \mathrm{d}\tau' \,\Gamma(L(\tau'),L_0,\partial L/\partial\tau')}.$$

Changing variable from cell cycle duration τ to division length L, we find the distribution of division length is

$$\mathrm{P}(L|L_0) = \frac{\mathrm{P}(\tau|L_0)}{(\partial L/\partial\tau)} = S(L, L_0)e^{-\int_{L_0}^L \mathrm{d}L' S(L',L_0)},$$

where we used Γ from Eq. 1 of the main text with $G(t) = 1$. A simple relation between the size control hazard $S(L, L_0)$ and the above is therefore

$$S(L, L_0) = \frac{\mathrm{P}(L|L_0)}{1 - \int_{L_0}^L \mathrm{P}(L|L_0)},$$

(S1)

or, equivalently,

$$S(x, L_0) = -\frac{\partial}{\partial x} \ln \mathrm{P}(L > x|L_0),$$

where

$$\mathrm{P}(L > x|L_0) = 1 - \int_{L_0}^{x} \mathrm{d}L\, \mathrm{P}(L|L_0) = e^{-\int_{L_0}^{x} \mathrm{d}L' S(L', L_0)}$$

is the the probability of reaching length x before dividing. Eq. S1 is a non-parametric estimator of the size control hazard in clock-deletion cells, which is shown in Fig. 3A, Supp. Figs. 4A and 11D-F. See Refs. (7, 8) for further information.

6.2 Size control hazards parameterized using linear models

In this subsection, we explain how to parameterize the dynamic model of the size control of the clock-deletion strain. We assume a linear model

$$\Delta_0 = L - (1 + a)L_0,$$

where Δ_0 follows a gamma distribution with shape and scale parameters $\beta_1$and β_2

$$p(\Delta_0) = \frac{1}{\Psi(\beta_1)\beta_2^{\beta_1}} \Delta_0^{\beta_1 - 1} e^{-\frac{\Delta_0}{\beta_2}},$$

where Ψ is the gamma function. The gamma distribution is a continuous distribution characterised by only two parameters, essentially describing mean and variance. For small variances, the Gamma distribution converges to the Gaussian distribution, but for larger variances it enjoys the particular advantage of being strictly positive (unlike a Gaussian distribution).

The size control hazard $S(\Delta_0)$ then follows from the division length distribution

$$\mathrm{P}(L|L_0) = p(L - (1 + a)L_0),$$

as

$$S(L, L_0) = S(L - (1 + a)L_0).$$

The above is obtained as the ratio between this distribution and its survival function, giving the relation

$$S(\Delta_0) = \frac{p(\Delta_0)}{\int_{\Delta_0}^{\infty} \mathrm{d}x\, p(x)}. \tag{S2}$$

Using the gamma distribution, this equation evaluates to

$$S(\Delta_0) = \frac{e^{-\frac{\Delta_0}{\beta_2}} \left(\frac{\Delta_0}{\beta_2}\right)^{\beta_1 - 1}}{\beta_2 \Gamma_{incompl}\left(\beta_1, \frac{\Delta_0}{\beta_2}\right)}, \tag{S3}$$

where $\Gamma_{incompl}$ denotes the upper incomplete gamma function.

6.3 Unbiased estimation of growth parameters

In principle, the cell size control parameters a, β_1 and β_2 can be found by estimating the slope a through a linear regression and evaluating the residuals of the regression. The residuals are are then fit to a gamma distribution to extract β_1 and β_2. However, the parameters obtained using this simple approach are typically biased when we do not account for unobserved growth before and after division. The power of the Bayesian approach is that we can estimate the true parameters given these uncertainties.

To this end, we assume cells are imaged every Δt and denote the length measurements in the birth and division frames as $L_{0,i}$ and L_i taken at times $t_{0,i}$ and t_i, for $i = 1,2,..,N$ respectively. The true birth and division lengths are

$$L_{b,i} = L_{0,i} \exp \int_{t_{0,i}}^{t_{0,i} - u_{1,i}} \mathrm{d}t' \alpha(t')$$

and

$$L_{d,i} = L_i \exp \int_{t_i}^{t_i + u_{2,i}} \mathrm{d}t' \alpha(t') ,$$

where $u_{1,i}$ and $u_{2,i}$ are unknown time-intervals in $[0, \Delta t)$. The growth rate in the different conditions are described in SI Sec. 9.

Assuming that the unobserved time-intervals are uniformly distributed in $[0, \Delta t)$,the likelihood of the growth parameters reads

$$\mathcal{L}(a, \beta_1, \beta_2 | \{L_i, L_{0,i}\}_{i=1,..,N}) = (\Delta t)^{-2N} \prod_{i=1}^{N} \int_0^{\Delta t} \mathrm{d}u_{1,i} \int_0^{\Delta t} \mathrm{d}u_{2,i}\, p(L_{d,i}(u_{1,i}, L_{0,i}) - (1+a) L_{b,i}(u_{2,i}, L_i)).$$

We sampled the posterior distributions of the growth parameters using an adaptive MCMC sampler (9) and evaluating the above integrals numerically. In Supp. Fig. 3, we demonstrate the

uncorrected regression method leads to biased estimates of the growth parameters, while the Bayesian method accounting for unobserved growth removes this bias.

7. Inference of the circadian coupling function from single-cell data

To determine the coupling function, we need to estimate its likelihood. The probability for a single cell born at time t_0 to divide after a cell cycle duration τ is a product of the probability of cell division $\Gamma(L(t_0+\tau), L(t_0), \frac{\partial L}{\partial \tau}, t_0+\tau)d\tau$ and the probability that the given cell has not divided before that time. The result is

$$\mathrm{P}(\tau|\mathbf{L}(t)_{t\in[t_0,t_0+\tau)}, \frac{\partial L}{\partial \tau}, t_0) = \Gamma(L(t_0+\tau), L(t_0), \frac{\partial L}{\partial \tau}, t_0+\tau)e^{-\int_0^{\tau} \mathrm{d}\tau' \,\Gamma(L(t_0+\tau'),L(t_0),\frac{\partial L}{\partial \tau'},t_0+\tau')}. \quad \text{(S4)}$$

This expression depends not only on the whole single-cell trajectory of length but also on its derivative with respective to cell age τ'.

Because derivatives are difficult to estimate from single-cell data, we focus on the likelihood for a cell to divide at a specific length, which can be obtained by a change of variable. Using Eq. 1 of the main text in Eq. S4, we obtain the probability of a cell to divide at a length L,

$$\mathrm{P}(L(t_0+\tau)|\mathbf{L}(t)_{t\in[t_0,t_0+\tau)}, t_0) = G(t_0+\tau)S(L(t_0+\tau)-(1+a)L(t_0))e^{-\int_{L(t_0)}^{L(t_0+\tau)} \mathrm{d}x \, G(t_0+\tau'(x))S(x-(1+a)L(t_0))}, \quad \text{(S5)}$$

a quantity that is independent of the length derivatives but depends on cell age $\tau'(L)$. Since cell length strictly increases in almost all of the observed single-cell traces, we estimated this quantity by interpolating cell age against cell length measurements for each cell using a cubic B-spline and evaluated the resulting integral in the above equation numerically.

The likelihood of the coupling function given N single-cell observations is then given by

$$\mathcal{L}(\mathbf{G}(t)_{t\in[0,24)}|\{t_{0,i}, \mathbf{L}_i(t)_{t\in[t_{0,i},t_{0,i}+\tau_i]}\}_{i=1,..,N}) = \prod_{i=1}^{N} \mathrm{P}(L_i(t_{0,i}+\tau_i)|\mathbf{L}_i(t)_{t\in[t_{0,i},t_{0,i}+\tau_i)}, t_{0,i}). \quad \text{(S6)}$$

Accounting for the unobserved growth (see SI Sec. 6 for details),

$$\mathcal{L}'(\mathbf{G}(t)_{t\in[0,24)}|\{t_{0,i},\tau_i\}_{i=1,..,N}) = \Delta t^{-2N} \prod_{i=1}^{N} \int_0^{\Delta t} \mathrm{d}u_{1,i} \int_0^{\Delta t} \mathrm{d}u_{2,i} \, \mathcal{L}(\mathbf{G}(t)_{t\in[0,24)}|\{t_{0,i}-u_{1,i}, \mathbf{L}_i(t)_{t\in[t_{0,i},t_{0,i}+\tau_i+u_{2,i}]}\}_{i=1,..,N}), \quad \text{(S7)}$$

where u_1 and u_2 are independent random variables that are uniformly distributed in the interval $[0, \Delta t)$. The unobserved growth was estimated by extrapolating the length-traces. We parameterized the coupling function $G(t) = \mathrm{Exp}(B(t))$, a positive function with arguments taken modulo 24 hours, by a cubic B-spline $B(t)$ with 12 knots (0,2,...,22 h) and periodic boundary conditions. The spline was evaluated at the knots and we sampled the posterior distributions using an adaptive Gibbs-sampler implemented in the Julia library Mamba (9). The result of this inference is shown in Fig. 3E.

Direct estimation of the coupling function is complicated because of the difficulty to enumerate all possible cell length histories. Alternatively, we can obtain an estimate of the division rate by simply ignoring the history-dependence in Eq. S5. By doing so, we may estimate the division length distribution

$$\mathrm{P}(L|L_0,t) = \int [\mathrm{d}\mathbf{L}(t)_{t\in(t_0,t_0+\tau)}]\mathrm{P}(L(t_0+\tau)|\mathbf{L}(t)_{t\in[t_0,t_0+\tau)},t_0)\mathrm{P}(\mathbf{L}(t)_{t\in(t_0,t_0+\tau)},t_0) \tag{S8}$$

and then compute the approximate size-control hazard

$$\frac{\Gamma(L,L_0,t)}{\partial L/\partial t} \approx \frac{\mathrm{P}(L|L_0,t)}{(1-\int_{L_0}^{L}\mathrm{P}(L|L_0,t))}, \tag{S9}$$

as shown in Supp. Fig. 5.

Note, however, that due to the average over histories in Eq. S8, which enter through the coupling function (cf. Eq. S5), Eq. S9 is a biased estimator of the size-control in the presence of circadian modulation. In contrast, in the absence of circadian modulation, the division length depends only the birth length and Eq. S9 equals the size control hazard S as explained in SI Sec. 6.1. The non-parametric estimator of the division rate is thus unbiased for clock deletion cells but not for WT cells.

8. Estimation of the circadian coupling function using division times (ignoring cell size control)

An alternative model for estimating the coupling function is to ignore the dependence on cell length in the division rate. The division rate $\Gamma(t,\tau)$ then depends only on the present time of day t and on cell age τ, Eq. S4 then reduces to

$$\mathrm{P}(\tau|t_0) = \Gamma(t_0+\tau,\tau)e^{-\int_0^{\tau}\mathrm{d}\tau'\,\Gamma(t_0+\tau',\tau')}.$$

In this simpler model, which we call the division-time model, the division rate can be obtained directly from inverting the above expression. The non-parametric estimator is given by

$$\Gamma(t_0+\tau,\tau) = \frac{\mathrm{P}(\tau|t_0)}{1-\int_0^{\tau}\mathrm{d}\tau'\mathrm{P}(\tau'|t_0)}. \tag{S10}$$

Note that the function in the denominator is just the probability that a cell born at time t_0 has not division before reaching age τ. In Supp. Fig. 4A,B, we show that the division rates clock-deletion and WT cells obtained using the above equation. Specifically, by plotting the ratio of the division rates (Supp. Fig. 4C), we find that Γ factorises as

$$\Gamma(t, t_0) = G(t)S(t - t_0),$$

where the functions G and S can be estimated efficiently using the likelihood

$$\mathcal{L}(\mathbf{G}(t)_{t\in[0,24)}|\{t_{0,i}, \tau_i\}_{i=1,..,N}) = \prod_{i=1}^{N} G(t_{0,i} + \tau_i)S(\tau_i)e^{-\int_0^{\tau_i} \tau' \, G(t_{0,i}+\tau')S(\tau')},$$

(S11)

which depends on the measured birth times and cell cycle durations $\{t_{0,i}, \tau_i\}_{i=1,\dots,N}$. Here, the birth time refers to the acquisition time of the first frame, and the cell cycle duration is the difference between the acquisition times of the first and last frames the cell was observed in. Since cells were imaged every Δt (45 min in constant light) and cell divisions were not directly observed, we accounted for the uncertainty in division times

$$\mathcal{L}'(\mathbf{G}(t)_{t\in[0,24)}|\{t_{0,i}, \tau_i\}_{i=1,..,N}) = \prod_{i=1}^{N} \int_0^{\Delta t} \mathrm{d}u_{1,i} \int_0^{\Delta t} \mathrm{d}u_{2,i} \mathcal{L}(\mathbf{G}(t)_{t\in[0,24)}|\{t_{0,i} - u_{1,i}, \tau_i + u_{1,i} + u_{2,i}\}_{i=1,..,N})],$$

(S12)

Where u_1 and u_2 are independent random variables that are uniformly distributed in the interval $[0, \Delta t)$. Samples from the posterior distribution were obtained using an adaptive MCMC sampler as shown in Supp. Figs. 4D, 6 and 12, where we parametrise $G(t)$ using a cubic B-spline with periodic boundary conditions as in SI Sec. 7. $S(t - t_0)$ is the hazard function of a Gamma distribution, whose parameters are estimated alongside $G(t)$.

9. Parameters used in stochastic simulations

For model simulations, we use $S(\Delta_0)$ given by the hazard of the gamma distribution (Eq. S3). Because $S(\Delta_0)$ is an increasing function, it is bounded by $S(L(t + \Delta t) - (1 + a)L_0)$. $G_{max} = 3$ as seen from Fig. 3E and $\alpha_{max} = 0.05$ h^{-1} in constant light (0.13 h^{-1} in 12:12 LD, 0.09 h^{-1} in 16:8 LD), compare also Supp. Figs. 7 and 10. In constant light, the mean exponential elongation rate oscillates in a circadian manner in WT (shown in Supp. Fig. 7). In clock-deletion cells, it is constant throughout the day with a mean of 0.048 h^{-1}. Under LD cycles we use

$$\alpha(t) = \begin{cases} A_{T_L} \sin\left(\frac{\pi mod(t-12,24)}{T_L}\right) & \text{if } 0 \leq mod(t-12, 24) \leq T_L, \\ 0 & \text{otherwise,} \end{cases},$$

where mod is the modulus (remainder after division), and $A_{12} = 0.13$ h^{-1}, $T_L = 12$ h in 12:12 LD, and $A_{16} = 0.09$ h^{-1}, $T_L = 16$ h in 16:8 LD cycles. The remaining parameters are summarised in the following table.

**Parameter**	**Constant Light**	**12:12 LD**	**16:8 LD**
a	-0.34<br>(-0.38, -0.26)	-0.015<br>(-0.107, 0.041)	-0.17<br>(-0.23,-0.10)
β_1	125.27<br>(114.61,128.52)	38.19<br>(34.90, 47.56)	44.67<br>(40.82, 51.39)
β_2	0.032<br>(0.031, 0.034)	0.084<br>(0.071, 0.090)	0.081<br>(0.073, 0.086)
Δt	0.75 h	1 h	1 h
σ	0.1	0.1	0.1

Parameters a, $\beta_1$and β_2 represent medians of the posterior distributions sampled using the method described in SI Sec. 6.3. Brackets denote the corresponding 95% credibility interval.

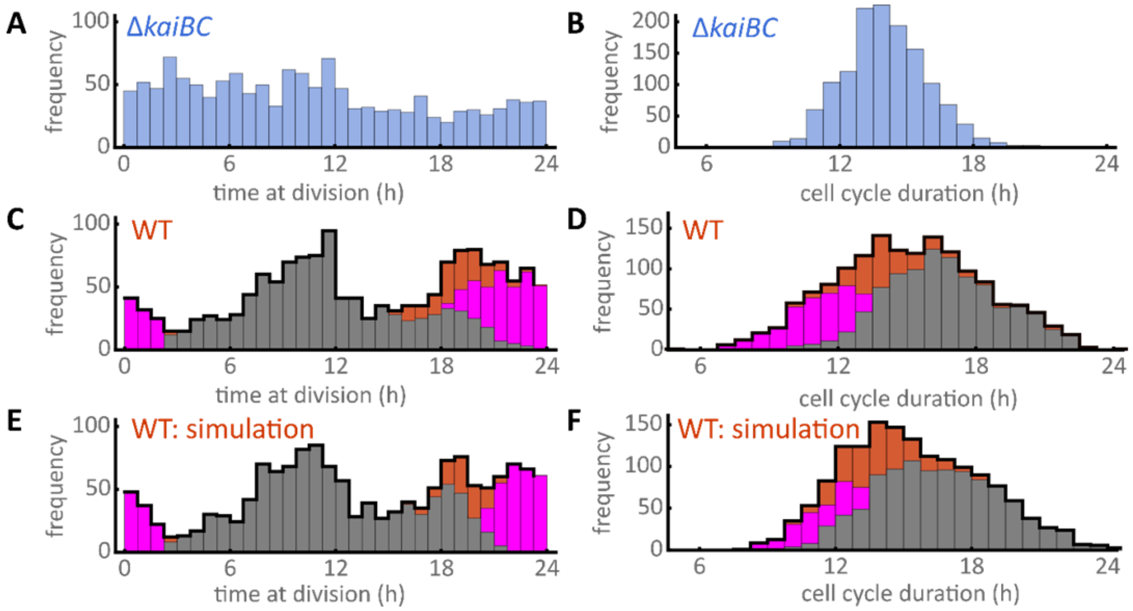

Fig. S1. Distribution of times at division and cell cycle durations in constant light. (A-B) Distribution of times at division and cell cycle durations in clock-deletion cells. Divisions occur uniformly throughout the day. **(C-D)** Times at division in WT cells are non-uniformly distributed, displaying a bimodal distribution (C, black line). The distribution of cell cycle durations is wider than in clock-deletion cells (D, black line). Clustering of subpopulations shows that fast cells (magenta histogram) divide mostly before dusk at the end of the subjective day while slow cells (grey histogram) divide at other times. **(E-F)** Distributions of times at division and cell cycle durations obtained from stochastic simulations recover the individual subpopulations. Sample sizes as in Fig. 2. Red histograms in (C-F) include all cells.

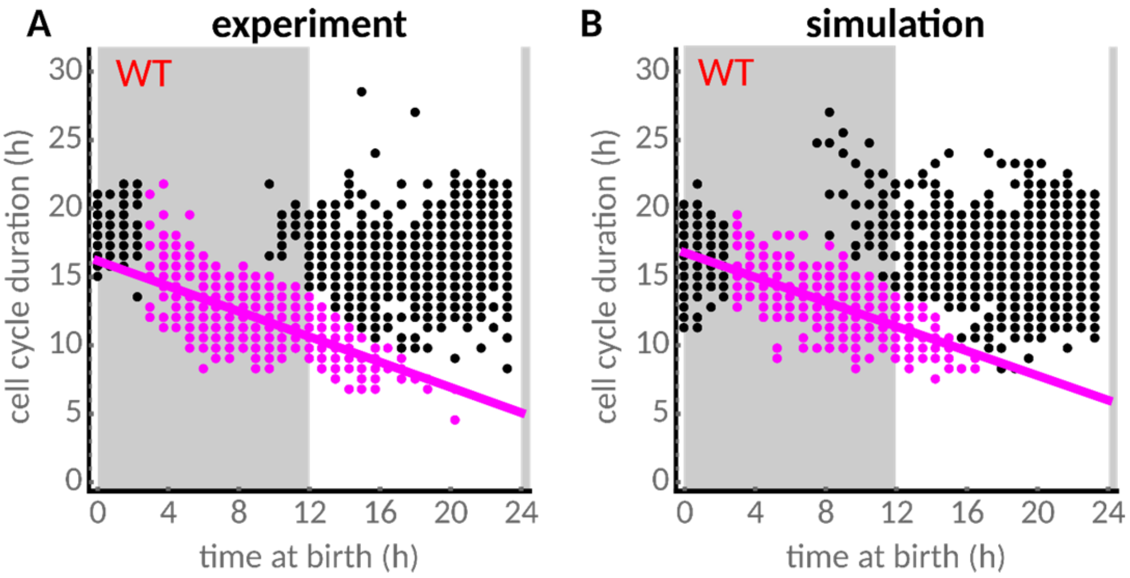

Fig. S2. Clustering identifies distinct subpopulations of WT cells in constant light and reveals an anti-correlation between time at birth and cell cycle duration. (A) Scatter of cell cycle durations and times of day at birth is clustered into two subpopulations using a Gaussian mixture model (SI Sec. 4). Cell cycle durations in the fast subpopulation (magenta dots) decrease with time at birth. **(B)** Clustering of simulation data is shown to recover slow and fast subpopulations and dependence of cell cycle duration on time at birth. Grey shades represent subjective night under constant light conditions.

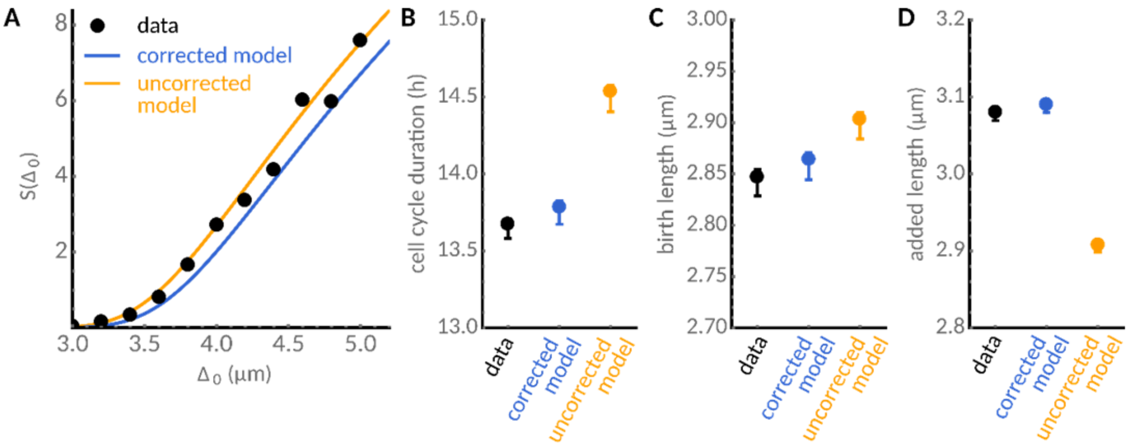

Fig. S3. Unbiased estimation of cell size-control parameters. Cell divisions occur in between measurements leading to unobserved growth, which biases size-control parameters. **(A)** Fit of cell size control hazard $S(\Delta_0)$ (yellow line) to bare experimental data (black dots) and the corrected estimate (blue line) taking into account unobserved growth, i.e., growth occurring between the last image before a cell division event and the first image after a cell division event. **(B-D)** In contrast to the uncorrected model, stochastic simulations using the corrected hazard yield unbiased predictions of (B) mean cell cycle duration, (C) mean birth and (D) division length, which agree well with the data within the error bars (95% confidence intervals obtained from bootstrapping).

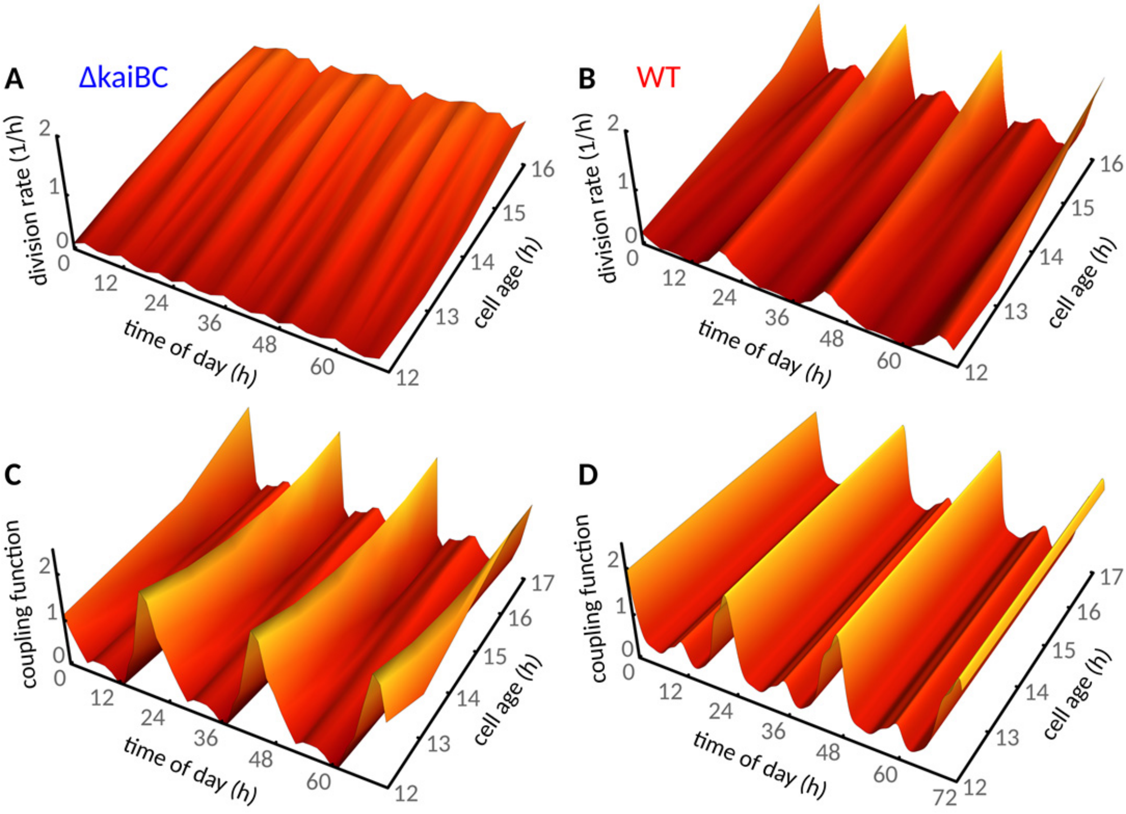

Fig. S4. Non-parametric estimations of the coupling function reveals no dependence on cell age, and is consistent with a Bayesian estimate. (A) Non-parametric division hazard for clock-deletion cells estimated directly from the data (SI Sec. 8, Eq. S10). **(B)** Equivalent non-parametric division hazard for WT cells. **(C)** Non-parametric coupling function obtained by the ratio of the WT to the clock-deletion hazard functions. **(D)** Bayesian inference of the coupling function from a minimal model that only depends on division times (SI Sec. 8, Eq. S12). Division rates are replicated for three cycles to highlight periodicity.

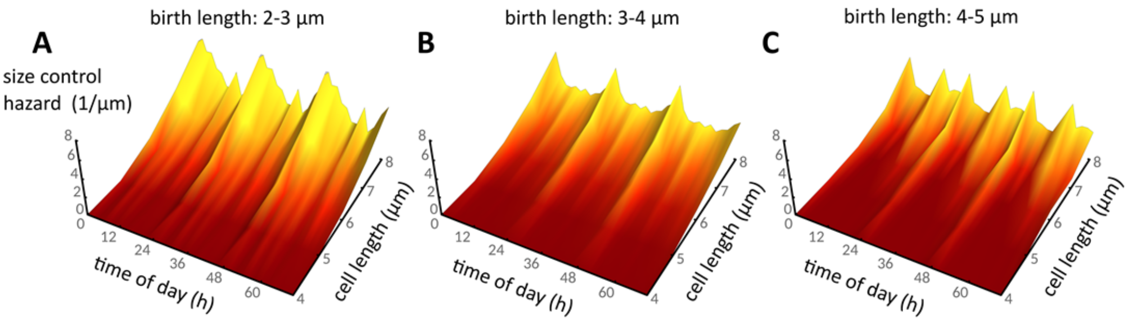

Fig. S5. Non-parametric estimation of the size-control hazard reveals circadian modulation of division rate in constant light. Size-control hazards for cell born with lengths **(A)** 2-3 µm, **(B)** 3-4 µm and **(C)** 4-5 µm. Size control hazards were computed by grouping WT cells depending on time of day at division and birth length, and using Eq. S9 in SI Sec. 7. Note that this direct estimation ignores some of the history dependence (see note in SI Sec. 7). For all three groups the hazard peaks towards the end of the subjective day independently of cell length. Hazards are replicated for three cycles to highlight periodicity.

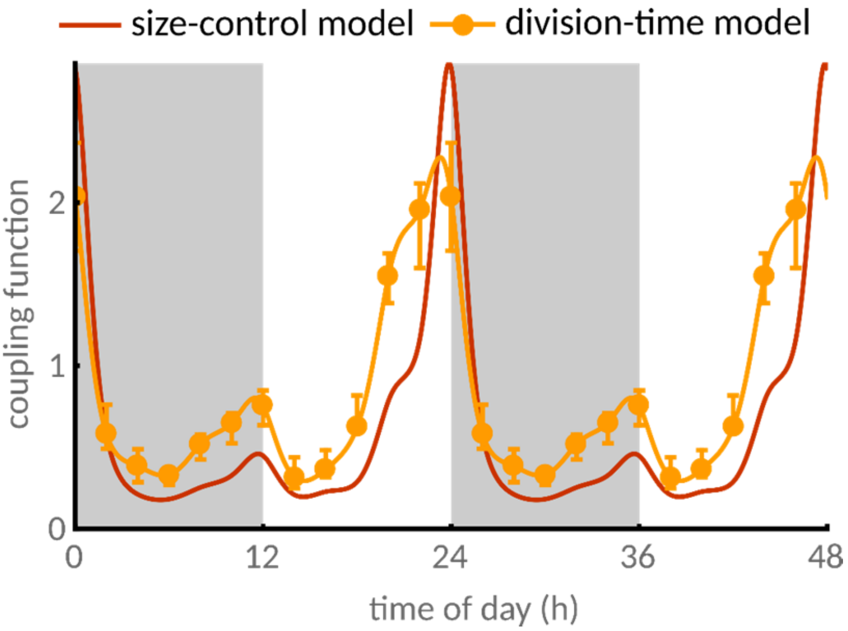

Fig. S6. Comparison of the circadian coupling function using size-control and division-time models. The division-time model (yellow points) uses birth and division times to infer the modulation of the division rate. The size-control model (red line) incorporates additional information about cell length traces (see SI Sec. 7 and SI Sec. 8 for details). The additional information about cell length narrows down the location of peak and shows stronger repression during the rest of the day. Coupling functions were parametrised by cubic splines (yellow and red lines, see also Fig. 3E). Grey shades represent subjective night under constant light conditions. Data replicated for two cycles to highlight periodicity.

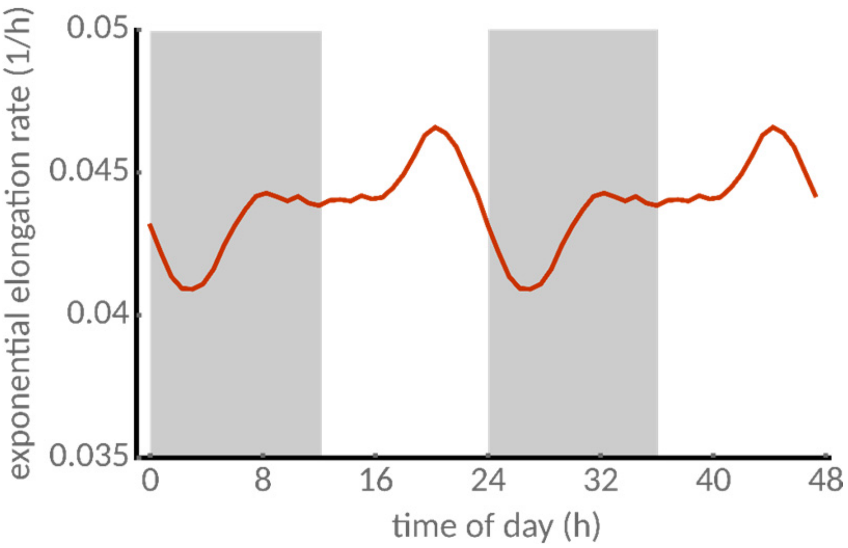

Fig. S7. Mean exponential elongation rate in constant light experiments. WT elongation rates oscillate throughout the day with a circadian period. Elongation rate peaks before subjective dusk and has a trough in early subjective night. Grey shades represent subjective night under constant light conditions. Data replicated for two cycles to highlight periodicity.

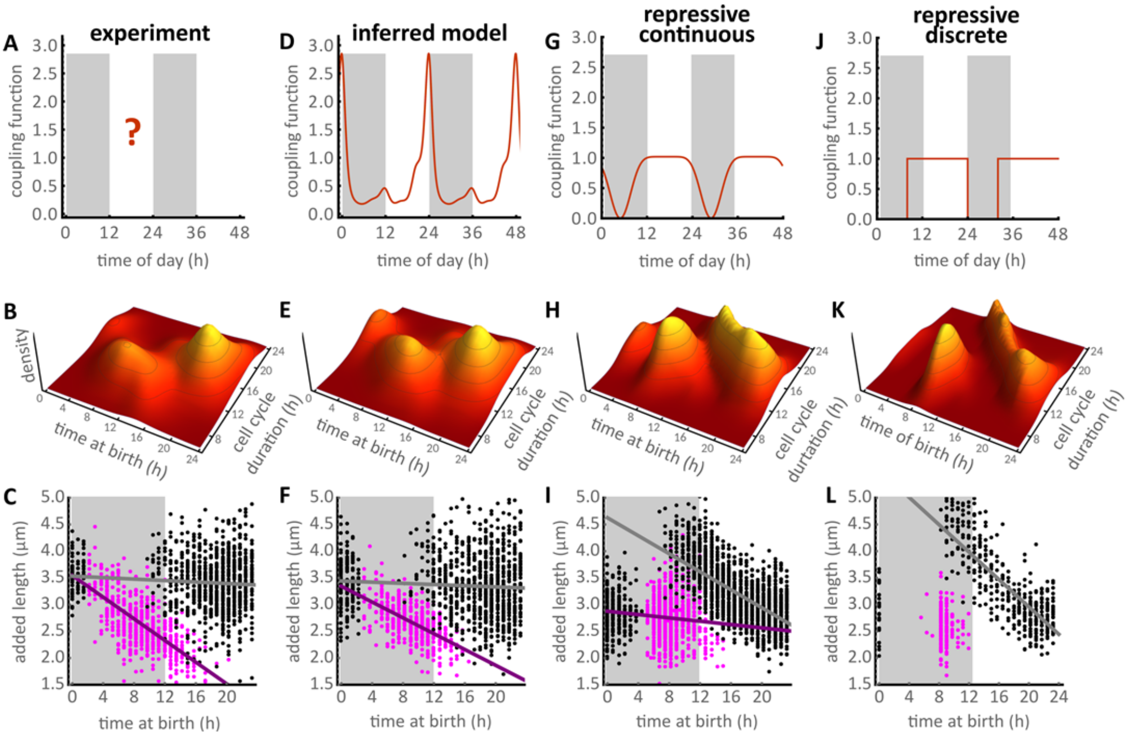

Fig. S8. Cell size data validates the coupling function. (A-C) Real coupling function is unknown. Experimental cell cycle durations display slow and fast subpopulations of cells born at different times of the 24-h day. Added length decreases throughout the day in the fast subpopulation (magenta dots) but not in the slow one (black dots). **(D-F)** Coupling function inferred in this study, which promotes cell divisions towards the end of subjective day. This coupling correctly predicts the dependence of added length on time of day at birth. **(G-J)** Two repressive couplings (on-off gates) proposed in the literature inhibit cell divisions in the beginning of subjective night. These couplings also generate two or three subpopulations but display decreasing added lengths in the slow subpopulations and a lower slope (violet lines) in the fast ones, which is not supported by our experiments (C). In the lower panels, magenta and black dots are single cell data points, and violet and grey lines are the regression lines for the fast and slow subpopulations, respectively. Grey shades represent subjective night. All panels show experiments or simulations for constant light conditions.

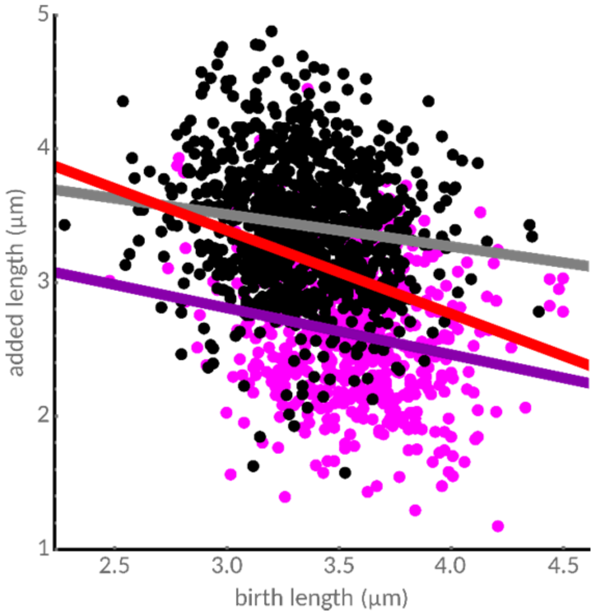

Fig. S9. Sizer-like principle in WT cells under constant light is a consequence of ignoring two subpopulations. The two subpopulations of cells (magenta and black dots) add different lengths. The slopes characterising the dependence of added length on birth length of each subpopulation (violet and grey lines with slopes -0.36 and -0.25, respectively) are less steep than the global slope (red line, slope -0.62). For comparison, the model predicts slopes of -0.30 and -0.24 in the fast (violet line) and slow (grey line) subpopulations respectively (as shown in Fig. 3H), which are well within the respective experimental 95% confidence intervals (-0.44, -0.27) and (-0.31, -0.17) obtained from bootstrapping.

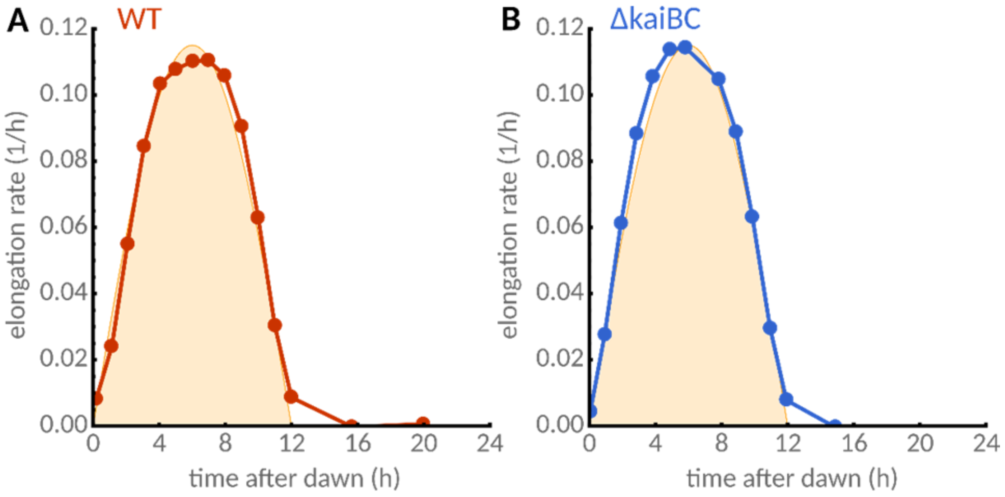

Fig. S10. Exponential elongation rates in 12:12 LD cycles. (A) The mean elongation rate of WT cells (red dots) in a 24 h period closely follows the imposed light profiles (yellow shade). **(B)** The mean elongation rate of clock deletion cells (blue dots) is nearly identical to the WT under these conditions and is also determined by the light profile.

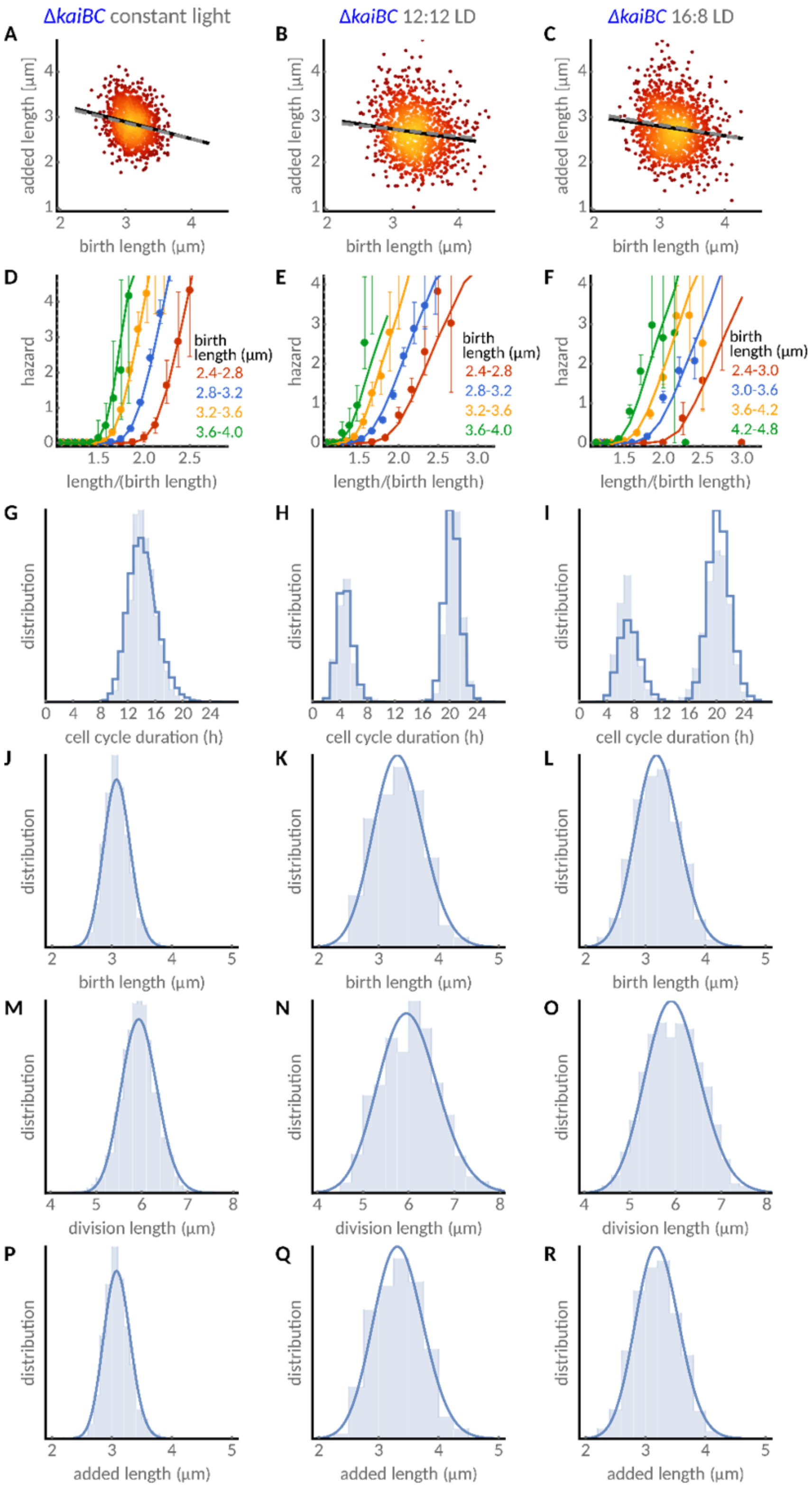

Fig. S11. Characterisation of cell size control and stochastic simulations in clock-deletion cells. (A-C) Stochastic simulations of single-cell density (dots) and linear fit (solid black line) in

constant light (1st column), 12:12 LD (2nd column) and 16:8 LD (3rd column) agree with slopes estimated from experiments (dashed grey line). **(D-F)** Size control hazards were estimated directly from the data for cells grouped by birth lengths (dots, see SI Sec. 6.1) and agree well with simulations (solid lines) in all conditions. **(G-S)** Comparison of experimental (light blue shades) and simulated (solid lines) single-cell distributions for cell cycle duration (G-I), birth length (J-L), division length (M-O), and added length (P-R) in all three conditions. See SI Sec. 9 for parameter values used.

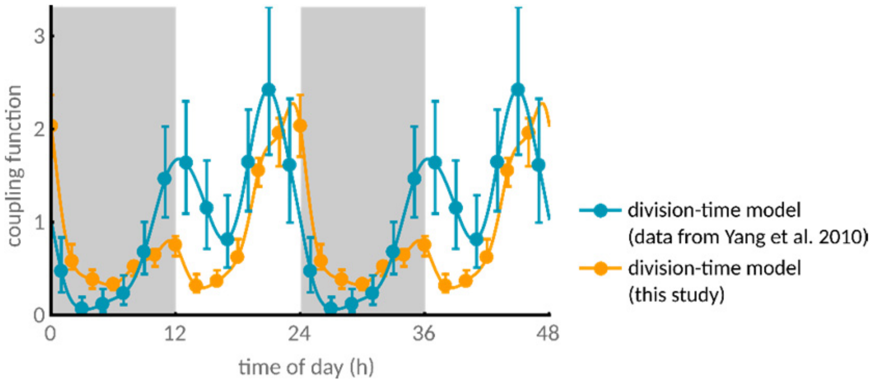

Fig. S12. Comparison between coupling functions obtained using data from this study and data from Yang et al. (10). We use the Bayesian approach (SI Sec. 8, Eq. S7) to extract the coupling function from division time data reported by Yang et al. We find the coupling function extracted from their data (teal) shares similar features with the coupling function found in this study (yellow), namely a peak of cell division towards the end of the day and lower division rates at other times. The coupling function from Yang et al. dataset (10) has been scaled to match the coupling function used in this study, which reports coupling levels relative to clock-deletion cells. Grey shades represent subjective night under constant light conditions. Data replicated for two cycles to highlight periodicity.

Table S1: Strains and plasmid used in this study.

S. elongatus strain	Description	Antibiotic resistance	Source
7942_A1	WT	None	ATCC
7942_A56	Δ*kaiBC mutant*	Gent	This study
**Plasmid**	**Description**	**Antibiotic resistance**	**Source**
pUC18-*kaiBC-Gent*	*kaiBC* deleted by the insertion of gentamycin cassette within the ORF	Gent	Teng *et al*. (3)

References

1. Young JW, et al. (2011) Measuring single-cell gene expression dynamics in bacteria using fluorescence time-lapse microscopy. Nat Protoc 7(1):80–88.
2. Johnson CH, Elliott JA, Foster R (2003) Entrainment of circadian programs. Chronobiol Int 20(5):741–774.
3. Teng S-W, et al. (2013) Robust Circadian Oscillations in Growing Cyanobacteria Require Transcriptional Feedback. Science 340(6133):737–740.
4. Nozoe T, Kussell E, Wakamoto Y (2017) Inferring fitness landscapes and selection on phenotypic states from single-cell genealogical data. PLoS Genet 13(3):e1006653.
5. Priestman M, Thomas P, Robertson BD, Shahrezaei V (2017) Mycobacteria modify their cell size control under sub-optimal carbon sources. Front Cell Dev Biol 5:64.
6. Thomas P (2018) Analysis of Cell Size Homeostasis at the Single-Cell and Population Level. Frontiers in Physics 6:64.
7. Osella M, Nugent E, Cosentino Lagomarsino M (2014) Concerted control of Escherichia coli cell division. Proc Natl Acad Sci U S A 111(9):3431–3435.
8. Kennard AS, et al. (2016) Individuality and universality in the growth-division laws of single E. coli cells. Phys Rev E 93(1):012408.
9. Smith BJ et al Mamba: Markov chain Monte Carlo for Bayesian analysis in Julia Available at: https://github.com/brian-j-smith/Mamba.jl.
10. Yang Q, Pando BF, Dong G, Golden SS, van Oudenaarden A (2010) Circadian gating of the cell cycle revealed in single cyanobacterial cells. Science 327(5972):1522–1526.
